# Chromogenic in situ Hybridization Compared with Real time Quantitative Polymerase Chain Reaction to Evaluate HER2/neu Status in Breast Cancer

**Published:** 2017-04-01

**Authors:** Hossein Ayatollahi, Azar Fani, Ehsan Ghayoor Karimiani, Fateme Homaee, Arezoo Shajiei, Maryam Sheikh, Sepideh Shakeri, Seyyede Fatemeh Shams

**Affiliations:** 1 *Cancer Molecular pathology Research center, Faculty of Medicine, Mashhad university of Medical sciences, Mashhad, Iran*; 2 *Solid tumor research center, Mashhad University of Medical Sciences, Mashhad, Iran*; 3 *Dept. of New Sciences and Technology, Mashhad University of Medical Sciences, Mashhad, Iran*

**Keywords:** CISH, Real-time qPCR, HER2, Breast Cancer

## Abstract

**Background and objective:**

The assessment of human epidermal growth factor receptor 2 (HER2) status has become of great importance in the diagnosis of breast cancer. The aim of this study was to investigate the diagnostic value of quantitative Polymerase Chain Reaction (qPCR) and Chromogenic In Situ Hybridization (CISH) to assess HER2 status of biopsy specimens.

**Methods:**

To elucidate the status of HER2 gene amplification, biopsies of breast carcinoma from 120 patients with 2+ IHC status were analyzed by qPCR and CISH.

**Results:**

The results of the two experiments were compared, and it was depicted that the concordance rate between CISH and qPCR assays was 88.1%.The quantification of HER2 gene with CISH and qPCR showed that there was a significant correlation (p value= 0.0001 and r= 0.808).

**Conclusion:**

The results of this research support the idea that qPCR is a precise and reproducible technique, which can be employed as a supplementary method to evaluate HER2 status.

## Introduction

The analysis of Human Epidermal growth factor Receptor 2 (HER2) has become of great importance in the diagnosis of breast cancer. Mutations of genes such as BRCA1, BRCA2, and p53 play an important role in the pathogenesis of this malignancy. Nevertheless, alterations in proto-oncogene pathways and tumor suppressor genes, including ERBB2/HER2, CD340, MLN19, NGL, NEU, and TKR1 may be involved in many types of human cancers (1). The ERBB2 gene (HER2 or neu) encodes a 185-kDa transmembrane glycoprotein with tyrosine kinase activity (2), which belongs to the epidermal growth factor receptor family. It has been found that HER2 protein is strongly associated with cell growth, differentiation, and survival (1, 3). 

The HER2/neu is located on the long arm of chromosome 17 (q21) and its amplification is reported in 20% to 30% of breast cancer cases, which is indicative of a poor prognosis (1, 4). The expression of HER2/neu might be a useful marker to predict response to cancer therapy, while it can also act as a candidate for therapeutic target gene (2). Amplification and expression of HER2/neu gene can be determined by several technique, such as Immunohistochemistry (IHC), Fluorescence in Situ Hybridization (FISH), Chromogenic in Situ Hybridization (CISH), and Real time Polymerase Chain Reaction (PCR), however; only CISH, FISH, and IHC have been approved by the US Food and Drug Administration (FDA) (1). Nevertheless, HER-2/neu expression can be obtained by CISH in paraffin-embedded samples (5). Chromogenic In Situ Hybridization is based on alkaline phosphatase, labeled reporter antibodies that are detected by an enzymatic reaction ^(5)^. It is also based on enzymatic detection and could be combined with IHC for the diagnosis of breast carcinoma. The most important advantage of the aforementioned method is the use of chromogens instead of fluorochromes for signal detection, which can be achieved with a standard bright field microscope (1).

It was recently reported that changes in both HER-2 gene copy numbers and amplification could be determined by PCR-based assays (6-9). The quantitative measurements of HER2 gene amplification are relatively new and highly sensitive assays that are improving in modern molecular pathology (10, 11). Dabbs et al. showed that there was a remarkable level of discordance between IHC and FISH results. In this study, high levels of false negative cases based on HER2 quantitative Real Time-Polymerase Chain Reaction (qRT-PCR) was shown, while these patients were reported HER2 positive, according to FISH analysis (12). More recently, literature reports have emerged that offer contradictory findings about HER2 analysis. Baehner et al. have suggested a concordance rate of 95% between qRT-PCR and FISH assay.

One of the most significant current discussions in the HER2 analysis is how to quantify the differences between available diagnostic assays. Moreover, there is an urgent requirement for guidelines regarding the appropriate use of confirmatory tests in addition to CISH or FISH. The experimental data are still rather controversial, and there is no general agreement on the use of qPCR for HER2 (7). In this study, we aimed to investigate the performance of the quantitative PCR assay in comparison with the CISH assay.

## Materials and methods

**Tumor material**

The study materials were breast cancer paraffin-embedded tissue samples with 2+ score for Her2/neu status in the IHC. Paraffin-embedded tumor tissue blocks were collected from 120 patients, processed, and stored at the department of molecular pathology of Ghaem hospital, Mashhad, Iran. Primarily, 3-µm sections of CISH and two sequential 10-µm thick sections were taken from the block for DNA extraction and transferred to a sterile 1.7-mL tube. To achieve accurate results, sections containing >80% tumor cells were selected for DNA extraction.

**DNA extraction**

DNA was extracted with the QIAamp DNA tissue reagent, according to the QIAGEN protocol. The concentration and quality of the DNA was analyzed with NanoDrop 2000 spectrophotometer (NanoDrop Technologies, Wilmington, DE, USA). A ratio of 1.8 was considered to indicate DNA purity.

**Real Time quantitative Polymerase Chain Reaction**

Real time qPCR analysis was performed using the Step One ABI detection system. All reactions were run in duplicates in separate wells that contained a 10-µL mixture; each reaction contained 0.5 g/L bovine serum albumin, 6 mM MgCl_2_, 0.5 µM of each primer, 0.2 µM of each hybridization probe, 0.2 mM of oxynucleotide triphosphate, and 0.5 U of Taq DNA polymerase in 1X PCR buffer, and 2 µL of DNA extraction at a concentration of 4 ng. The PCR program started with one cycle at 95**°**C for 30 seconds, followed by 50 amplification cycles at 95**°**C for 3 seconds, 55**°**C for 5 seconds, and 72**°**C for 10 seconds. The 5’- and 3’-end nucleotides of the probe were labeled with the reporter 6-Carboxy-Fluorescein (FAM) and the quencher dye 6-Carboxy-Tetramethylrhodamine (TAMRA), respectively. All reactions were performed in an ABI prism 7700 sequence detection system (Applied Biosystems AB, USA). The primers and probes used in this analysis are presented in Table 1. The content of the target in tumor samples was quantified using standard curves to determine a relative measure for the initial amount. The absolute target copy numbers were resolute with utilization of 1:2 dilution series of genomic DNA, as the control gene standard. For each clinical sample, the amounts of the target gene (HER-2/neu) and the reference gene (IGF-1) were calculated in tumor tissue and healthy control tissue. The sequence of primer and probes and instruction of the protocol has been previously described (8). According to the previously published data, a ratio between HER2 and reference gene of less than <2 indicates that the patient sample has negative results for HER2 amplification while a ratio of greater than >2 indicates that the patient sample has positive results for HER2 amplification (3, 6, 13).

**Table 1:** Sequence of Probe and Primer Used in This Study

Target	Name ^a^	Sequence, 5´ - 3´
HER-2/neu	neu-F	GAACTGGTGTATGCAGATTGC
	neu-R	AGCAAGAGTCCCCATCCTA
Probe	Neu-up	GTATGCACCTGGGCTCTTTGCAGGTCTCT-FAM
	Neu-down	LCRed640 CCGGAGCAAACCCCTATGTCCACAAGG-p
IGF-1	IGF-F	AGCTCGGCATAGTCTT

**Chromogenic In situ Hybridization (CISH)**

The CISH assay for analysis of HER2/neu gene was performed according to the manufacturer’s instructions (ZytovisionGmbh, Fischkai, Germany), as follows: Following deparaffinization, the tissue was placed in heat pretreatment solution (reagent A-1) at 95^o^C for 15 minutes. After a wash with deionized or distilled water (dH_2_O), the enzyme solution (reagent A-2) was added for 5 minutes at room temperature, following the wash with dH_2_O. After dehydration in ethanol and air-drying the section, the probe (reagent A-3) was added and overnight hybridization was performed at 37^o^C. Slides were then incubated in washing buffer (reagent B-1) at 75^o^C to 80^o^C for 5 minutes. After rinsing in distilled water, Anti-DIG/DNP-Mix (reagent B-2) was applied for 15 minutes at 37^o^C. The slides were then washed in Tris-Buffered Saline (TBS) (reagent B-3) and HRP/AP-Polymer-Mix was applied for 15 minutes at 37^o^C in the humidity chamber. Next, one drop of AP-Red was added to the solution (reagent B-4) and incubated for 10 minutes at room temperature, followed by addition of HRP-Green solution (reagent B-4) and further incubation for 10 minutes at room temperature. Slides were then counterstained with hematoxylin and dehydrated, and covered by a cover slip. The CISH signals were evaluated with a bright microscope with a 40×dry objective lens. Results were marked as negative by CISH if a median ratio of HER-2/CEP17 was less than <2.2, while a ratio greater than 2.2< was considered as a positive result.

**Statistical analysis**

Data analysis was done by SPSS statistical software version 11.5 (SPSS Inc., Chicago, IL, USA). Comparisons between HER-2 expression (qRT-PCR) and gene copy number (CISH) were calculated using the Chi square test. The correlation between the two methodologies was evaluated using One-way Analysis of Variance (ANOVA); differences were tested for significance by the Mann-Whitney test for two categories. Statistical significance was considered at P < 0.05. 

## Results

**Population Characteristics**

 In this study, 120 females with non-inflammatory breast cancer, and age range of 29 to 79 years old (mean age 49), were examined. Furthermore, 120 Formalin-Fixed Paraffin-Embedded (FFPE) breast carcinoma samples were examined by qPCR and CISH. Tumor cellularity was analyzed and the mean value was 83.2%. In cases with negative HER2 test results, the median ratio of HER2 status, based on the CISH assay, was 1.12 (range 1-1.5). Among cases with positive results, a ratio greater than 2.2 was observed in 45 (37.5%) cases.

**Comparison of HER-2 Gene Amplification by** **Chromogenic in Situ Hybridization** **and Quantitative Polymerase Chain Reaction**

**Figure 1:**
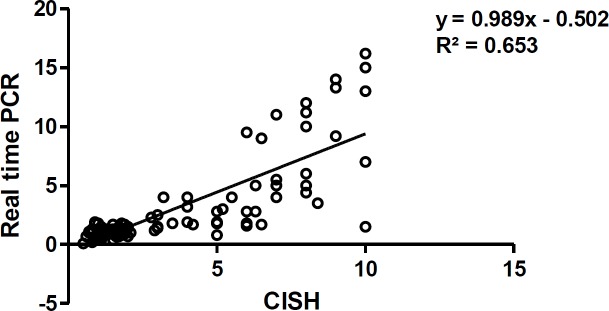
Quantification of HER-2 Gene with Chromogenic in Situ Hybridization and Real-Time Polymerase Chain Reaction Indicating a Significant Correlation (p value= 0.0001 and r= 0.808)

The HER2 gene amplification was detected in all 120 samples by CISH and qPCR assays. There was no amplification in 75 (62.5%) out of 120 samples, and amplification of HER2 gene in 45 (37.5%) out of 120 samples was achieved by the CISH method, while in 31 (25.8%) out of 120 specimens amplification of HER-2 was achieved by qPCR. The concordance rate between real-time qPCR and CISH was 88%. In addition, 31 (68%) out of 45 samples with CISH positive test results demonstrated a ratio greater than 2 by qPCR technique. Discrepancies were found in only 14 samples, which were amplified by CISH and not amplified with real-time PCR. The calculated HER-2/reference gene ratio was significantly higher in specimen with CISH positive test results. The quantification of the amplified HER2 gene with CISH and real-time PCR showed a significant correlation (Figure 1) (p value= 0.0001 and r= 0.808).

**Figure 2:**
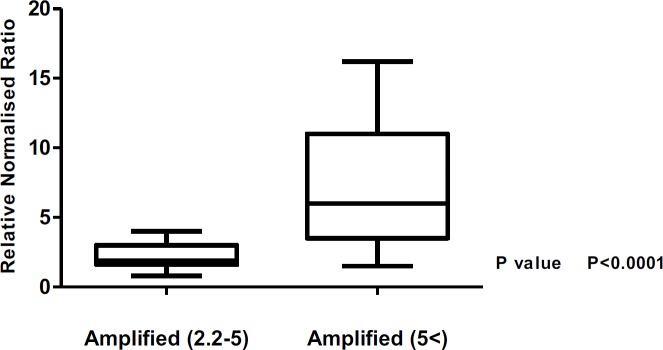
Quantitative Polymerase Chain Reaction Amplification Analysis in HER2 Amplified and Non-amplified Breast Cancer Specimen

The qPCR amplification analysis in HER2 with amplified and non-amplified breast cancer specimen showed that in both groups the logarithmic Relative Normalized Ratios (RNR) was significantly different from the P value of <0.0001. The mean values ± Standard Error of the Mean (SEM) in the amplified HER2/CISH group and the non-amplified group was 6.624 ± 0.72 and 1.075 ± 0.044, respectively (Figure 2).

The HER2 status was assessed in 44 FFPE amplified samples. Firstly, two sub-groups were divided based on their CISH scores (2.2 to 5 and >5). Then, the comparison of HER2 amplification, following the genomic DNA qPCR analysis, depicted a significant difference (P value<0.0001) between the two sub-groups of HER2 positive patients with scores of 2.2 to 5 (17 cases) and >5 (27 cases).

Fold changes comparison in both groups showed that the logarithmic Relative Normalized Ratios (RNR) was significantly different from the P value of <0.0001.

## Discussion

Amplification of HER2/neu gene has been observed in 10% to 35% of the human breast cancer patients (14). In addition, for better management of breast cancer, to analyze the HER-2 status it is essential to choose the best treatment strategy. Patients with HER2/neu gene over-expression may experience adverse effects with anti-hormonal therapy (15-19). Moreover, trastuzumab (Herceptin, Genentech) is essentially useful for patients with amplified HER2, however, patients without amplification of HER2 gene show limited benefit from anthracycline ^[20, 21]^. Immunohistochemistry (IHC) techniques are commonly used for determining HER2 status; their results in cases presenting negative (0 or +1) or positive (+3) expression or those showing moderate protein expression (+2) must be evaluated by other methods such as the Fluorescence In Situ Hybridization (FISH) assay (22). Recently, CISH has improved as a powerful alternative method to FISH for confirming moderate IHC results. Other studies have shown favorably validated CISH results (23, 24). The concordance between CISH and FISH ranged from 95% to 100% (23, 25, 26) and allows selection of fields of invasive ductal carcinomas. Nevertheless, CISH is easier for pathologists to interpret protein expression with detailed morphologic features of tumors. Another advantage compared to CISH is its cost effectiveness and the ability to be performed using a light microscope (5). 

Real-time PCR has become a robust technique with higher speed and automation. Quantitative PCR is a relatively easy assay to detect small amounts of DNA that maybe used in Formalin-Fixed Paraffin-Embedded (FFPE) specimen (10, 11). In this study we therefore determined HER-2 gene amplification results by real-time PCR and CISH assay in parallel. The results of this study indicate that there is 88% concordance between qPCR quantification assay and CISH. It is well comprehended that under the best circumstances in a high quality molecular laboratory, measurement of the same target by two different techniques will definitely lead to discordant data. Moreover, in the HER2 status analysis, there has been no definite gold standard for response to targeted therapy.

In the present study, the analysis of HER2 status based on the CISH method revealed that 37.5% of FFPE specimens were above the cut-off value (ratio ≥ 2.2). In addition, 14 samples out of 45, were HER2 negative with qPCR test but were amplified with CISH as a current standard method. The mean ratio of these 14 samples (1.89, n=14) was very close to the cut-off value of 2.00, while all the remaining positive samples had a ratio of > 2 (range: 2.3 to 16.02, median =6.77, and n=31). The majority of the samples (31/45, 68%) that were classified as positive by qPCR, had also been positive by CISH analysis. In 14 samples, HER2 gene amplification was present following CISH analysis, while no amplification was observed using qPCR.

There was understandable disagreement between diagnostic approaches that assess the HER2 status. Moreover, biologists should support the idea that a robust and accurate approach with improved standards may be implemented at any molecular diagnostic laboratory (13, 14). 

In the field of breast cancer molecular assessment, increasing numbers of targeted therapeutic regimen are being developed, which are not confined to the HER2 pathway. However, according to the results of the current study, CISH and qPCR may be equally valued, as these techniques are crucially important in genetic-related analysis of HER2. Perhaps patients may benefit when a novel molecular technique is developed than when existing methodologies are improved. 

Creation of an additional quantitative exam to the routine so called “gold standard” for HER2 will significantly reduce the number of cases, who had been misdiagnosed and consequently mismanaged for their targeted therapy against the oncogene. Therefore, the implementation of additional diagnostic tools such as qPCR to the CISH assay will definitely improve the disease outcome and also the response to therapy in considerable number of cases. Undoubtedly, these are challenging concepts and scientists are still not capable of measuring all biomarkers with multiple techniques consistently (27). 

Furthermore, many diagnostic tests have appropriate quality, which is why supplementary research may not make dramatic improvement to the quality of these approaches. In this regard, to enhance the diagnostic value of the detection assays of HER2 or even to create a novel assay will be extremely useful for breast cancer patients with ambiguous results after either IHC or FISH. There is abundant space for further progress in determining HER2 amplification patterns, including cases with heterogeneous amplification. Therefore, further research should be done to improve accurate and informative HER2 testing. 
